# Marine Actinomycetes, New Sources of Biotechnological Products

**DOI:** 10.3390/md19070365

**Published:** 2021-06-25

**Authors:** Sveta V. Jagannathan, Erika M. Manemann, Sarah E. Rowe, Maiya C. Callender, William Soto

**Affiliations:** Department of Biology, College of William & Mary, Williamsburg, VA 23185, USA; svjagannathan@email.wm.edu (S.V.J.); emmanemann@email.wm.edu (E.M.M.); serowe@email.wm.edu (S.E.R.); mccallender@email.wm.edu (M.C.C.)

**Keywords:** microbial experimental evolution, industrial microbiology, actinomycetes

## Abstract

The Actinomycetales order is one of great genetic and functional diversity, including diversity in the production of secondary metabolites which have uses in medical, environmental rehabilitation, and industrial applications. Secondary metabolites produced by actinomycete species are an abundant source of antibiotics, antitumor agents, anthelmintics, and antifungals. These actinomycete-derived medicines are in circulation as current treatments, but actinomycetes are also being explored as potential sources of new compounds to combat multidrug resistance in pathogenic bacteria. Actinomycetes as a potential to solve environmental concerns is another area of recent investigation, particularly their utility in the bioremediation of pesticides, toxic metals, radioactive wastes, and biofouling. Other applications include biofuels, detergents, and food preservatives/additives. Exploring other unique properties of actinomycetes will allow for a deeper understanding of this interesting taxonomic group. Combined with genetic engineering, microbial experimental evolution, and other enhancement techniques, it is reasonable to assume that the use of marine actinomycetes will continue to increase. Novel products will begin to be developed for diverse applied research purposes, including zymology and enology. This paper outlines the current knowledge of actinomycete usage in applied research, focusing on marine isolates and providing direction for future research.

## 1. Introduction

Actinomycetales is an order of Gram-positive bacteria consisting of both benign and pathogenic bacteria belonging to the phylum Actinobacteria [1]. Actinobacteria have historically been characterized by high GC content in their DNA [2], though some members, particularly freshwater-dwelling ones, have been found in recent years to have relatively low GC content [3]. Members of this order are often distinguished by their mycelial morphology with branched hyphae and the ability to form spores, although not all actinomycetes are sporulating [4]. They exhibit great diversity in a variety of characteristics including moisture tolerance [5], habitat, optimal pH, and thermophilicity [4]. Actinomycetes are often found at moderate pH levels [6,7], though some acidophilic and alkaliphilic species are known [5,8,9,10]. While some thermophilic actinomycetes have been recorded [11], most species appear to prefer moderate temperatures [12,13]. This diversity is also reflected in the near ubiquity of actinomycetes in the environment [14,15,16,17], with samples having been discovered in remote locations such as the Mariana Trench [18] and Antarctica [19]. Actinomycetales diversity may even be greater than previously estimated, as flaws in traditional methods of bacterial classification such as 16S rRNA comparison may have led to errors in Actinomycetal identification [20,21,22]. Actinomycete species are primarily found in soils [2] and were originally thought to be solely terrestrial. This belief was supported by the observation that some terrestrial actinomycete spores wash into the sea, which was thought to explain their presence in water samples [23]. In fact, the first discovery of a marine actinomycete was not until 1984 [24]. Since then, many marine species have been discovered in aquatic systems worldwide [25,26,27,28,29] and even single species have been shown to have widespread distribution in the world’s oceans [27,30].

Marine actinomycetes associate with a variety of aquatic organisms, including invertebrates such as sponges [27,31], corals [32,33], and echinoderms [7], as well as vertebrates such as pufferfish [34]. These interactions may encourage unique chemical ecologies that might influence the evolution of secondary metabolic pathways. In addition to associating with other organisms, marine actinomycetes can exist in both planktonic and biofilm niches, although the majority of strains have been isolated from sediments [35]. The characteristics that promote and result from these different life strategies are not yet well understood. General studies of planktonic and biofilm-forming bacteria indicate that these communities differ in species composition [36,37]. Population sizes of actinomycetes in ocean sediment have been shown to vary with physico-chemical parameters including temperature, pH, pressure, total organic carbon, and salinity, the preferred levels of these parameters varying with location. Strains including *Streptomyces*, *Micromonospora*, and *Actinomyces* have been found at depths as great as 500m [26]. *Micromonospora* in particular may have greater relative abundance at 450 m than at shallower depths [38]. In addition, studies have shown greater heat resistance of actinomycete samples collected from marine sediment than from seawater, leading to the hypothesis that the more heat-resistant spore form of actinomycetes predominates in sediment compared to the vegetative form [39]. Comparatively little research has been conducted on planktonic actinomycetes in comparison to those in sediments. Early evidence suggests that some planktonic strains are non-sporulating [29] and vary in their temperature optima [40], indicating that they fill a diversity of ecological niches. The relative dearth of discovered planktonic varieties may in part reflect problems with sampling, as bacterial abundance is affected by temporal variations in nutrient availability that are caused by geological activity [41]. Free-floating bacteria may also be more vulnerable to predation by grazers [42] and infection by marine viruses, which are widespread in ocean waters. Populations of rare bacteria may be especially depressed by the presence of viruses, hindering detection efforts [43]. In contrast, bacterial communities in sediment may gain some protection against infection as viral adsorption to sediment may reduce phage replication rates [44].

Although the ocean presents a vast and varied environment for bacterial populations, most microbiological research has focused on samples from terrestrial environments [45]. This trend may in part be due to greater difficulties in sampling and culturing microbes from seawater and ocean sediment [46]. However, marine and terrestrial environments differ substantially. Marine microbes develop adaptations not present in their land-based counterparts [38,47]. The need for novel adaptations suggests that marine actinomycetes possess unique metabolic and genetic characteristics that are a promising subject for future research. This biological potential is especially important given that actinomycetes have been exploited for decades as a source of bioactive compounds, especially antibiotics. *Streptomyces* species alone produce over 7600 bioactive microbial metabolites [48], with an increasing proportion of new metabolites being discovered from rare actinomycetes. As much initial research focused on soil-derived species, these have mostly been exhausted as a source of easily detectable compounds. In contrast, marine actinomycetes are only beginning to be characterized and exploited for bioactive compounds [49], and much of their species diversity remains unexplored. Even closely related strains possess unique biosynthetic gene clusters (BGCs) [50,51], groups of genes in proximity to each other on the chromosome that encode secondary metabolites [52]. The existence of these BGCs in close marine relatives suggests the continued investigation of metabolic potential will lead to the discovery of additional natural products that are important, useful, and valuable.

Despite the plethora of BGCs harbored by marine actinomycetes, the genetic diversity discovered by genome sequencing is not fully reflected in laboratory attempts to elicit secondary metabolite production. This relative sparsity of obtained secondary metabolites is due in part to cryptic gene clusters that are only activated under certain conditions [53]. For example, because marine actinomycetes often live in close proximity to other microorganisms, they may possess secondary metabolites for purposes of chemical defense that are only expressed in the presence of competing microbial strains [54]. In addition, the production of some antimicrobial compounds is enhanced by the presence of seawater [39,55,56], implying the existence of unique secondary metabolic pathways not present in terrestrial actinomycetes. To fully exploit the metabolic capacities of marine actinomycetes, new technologies and techniques to increase BGC expression must be utilized [57]. Strategies include the optimization of fermentation conditions [10], as well as advanced techniques such as pathway engineering [58] and gene cloning [59]. Despite the technological demands of natural product discovery, this method continues to promote significant development of novel drugs [60]. In contrast, synthetic approaches have yielded few approved compounds [61]. A final challenge lies in the selective isolation of rare actinomycete strains for further investigation and characterization [62]. Other potential targets for the sustained search of secondary metabolites are plasmids, which are well-documented in marine environments [63]. As in many bacteria, plasmids are common in actinomycete species, with some strains possessing a multitude [64]. Although actinomycetes may possess either linear or circular chromosomes [65,66], their plasmids are usually linear [64]. These extrachromosomal DNA elements can harbor genes encoding secondary metabolites similarly to BGCs [67,68], helping the host better adapt to its environment.

Some of the most extensively researched Actinomycetales are members of the genus *Streptomyces*. *Streptomyces* spp. are saprophytic bacteria found in soil as well as aquatic environments, which possess a variety of morphological forms that often resemble fungi [69]. Under unfavorable conditions, aerial hyphae extend away from the mycelium to release spores for dispersal [70]. Most importantly, *Streptomyces* spp. are the source for the majority of antibiotics discovered from Actinomycetales [48] and possess significant potential for new natural product discovery. It has been predicted that *Streptomyces*’ capacity for antibiotic production is on the order of 10^5^ and that increasing screening efforts would lead to an improved rate of antimicrobial discovery [71]. Whole-genome sequencing has revealed that individual *Streptomyces* strains can contain as many as 34 BGCs with secondary metabolite potential, many of which are yet to be explored as sources of antibiotics [72,73]. Due to their ubiquity and reliable history of secondary metabolite discovery, much actinomycete research has focused on members of *Streptomyces*. However, the study of rare actinomycetes is gaining popularity in an attempt to address the problem of natural product rediscovery [74]. As researchers exhaust the readily isolable products of common species, rare strains provide an alternative resource for the identification of novel secondary metabolites.

While marine actinomycetes are renowned as sources for the acquisition of antibiotics, they are also responsible for the production of other medically important compounds. The gene clusters responsible for these drugs are thought to partially originate from lateral gene transfer [72,75]. Some of these drugs have anticancer effects [76,77]. Coral-associated actinomycetes have shown the ability to inhibit the formation of biofilms [32,78], including those formed by antibiotic-resistant *Staphylococcus aureus* [79,80], which cause infection via medical devices. Some actinomycetes can also form biofilms that allow them to degrade complex polymers in the environment [81,82,83,84], indicating their value in composting or bioremediation efforts, especially toxic pesticides [85,86]. Actinomycetes are also being investigated for their ability to improve agricultural productivity [87,88]. The myriad functional capacities of actinomycetes may reflect their vast repertoire of secondary metabolic pathways, a survival advantage that granted them significant importance in the search for novel bioactive compounds.

## 2. Medical Applications

Over 80% of all antibiotics used in the medical field originate from Actinobacteria [89], with 50% of clinically relevant antibiotics originating from the *Streptomyces* [90]. Each actinobacterial strain has the potential to produce 10 to 20 secondary metabolites [91], reaffirming the phylum’s profound capacity to produce antibiotics. The primary drug classes for clinical antibiotics are aminoglycosides, β-lactams, glycopeptides, macrolides, and tetracyclines [92]. Specific antibiotics derived from actinomycetes that are used in clinics today include neomycin, streptomycin, kanamycin, cephamycin, vancomycin, erythromycin, and tylosin [2]. Here, we present a limited representation of actinomycete clinical applications.

### 2.1. Antibiotics

The development of antibiotics using novel secondary actinomycetal metabolites has been helpful for treating microbial infections in humans [2]. Rifampicin and cycloserine are antitubercular antibiotics used to treat *Mycobacterium tuberculosis* [93]. Respiratory infections, including Legionnaires’ disease, are treated with erythromycin, which was isolated from *Saccharopolyspora erythraea*. Tetracycline was isolated from *Streptomyces aureofaciens* and targets bacterial ribosomes. Daptomycin, derived from *Streptomyces roseosporus*, is typically used to treat vancomycin-resistant MRSA infections [94,95]. Chloramphenicol, from *Streptomyces venezuelae*, inhibits bacterial protein synthesis [96]. *S. aureus*, *Streptococcus* spp., and *Pseudomonas* spp. are susceptible to chloramphenicol [97]. Gentamicin, originated from *Micromonospora purpurea*, treats a range of infections caused by Gram-positive and Gram-negative bacteria [98]. Monensin, isolated from *Streptomyces cinnamonensis*, treats Gram-positive infections resistant to many other antibiotics [99]. There is still tremendous potential for discovering new clinical antibiotics by isolating novel actinomycete agents. Three novel angucycline nocardiopsistins were isolated from marine sponge-derived *Nocardiopsis* sp. with anti-MRSA activity [100]. In a study of twenty strains of actinomycetes, from the mangrove ecosystem of the Andaman Islands, thirteen strains showed broad-spectrum activity against Gram-positive and Gram-negative bacteria. Of the thirteen strains, one novel strain of *Streptomyces* sp. produced bioactive metabolites with broad-spectrum activity against fish, poultry, and fungal pathogens.

A frequently overlooked research area is subinhibitory concentrations of antibiotics, which are concentrations below the minimum inhibitory and minimum microbiocidal levels [101,102]. Subinhibitory concentrations of antibiotics can have deleterious or toxic effects on a pathogen or parasite that differ from those that occur at higher concentrations. For example, gene expression and regulation of particular genetic regulons, physiological networks, or metabolic pathways of pathogens can be disrupted or altered by antibiotics at subinhibitory concentrations. These disruptions or alterations can be quite distinct from the primary mode of action that an antibiotic has on its main target [101,102]. An interesting topic is whether or not the antibiotics produced by actinomycetes have effects on other microbes that are unique at subinhibitory concentrations, when compared to antimicrobial compounds produced by other organisms. For instance, subinhibitory antibiotic concentrations mediate nutrient use and competition among dissimilar strains of *Streptomyces* that reside in terrestrial habitats [103]. How do these results compare with subinhibitory levels of antibiotics produced by other organisms, such as plants and fungi? Additionally, are there differences among terrestrial, freshwater, and marine actinomycetes in the effects that their antibiotics have at subinhibitory concentrations, even when considering the same class of antibiotics?

### 2.2. Antibiotic Resistance

Despite the hundreds of antibiotics currently available, there is still potential and demand for the discovery of new antimicrobials. A decline in FDA-approved antibiotics since the “Golden Age” of antibiotic discovery, during the 1950s and 1960s, emphasizes the need for novel antibiotics [104]. Unfortunately, the development of new antibiotics is a slow process. Pharmaceuticals typically take 10 to 15 years to progress from initial molecule discovery to final drug development. Additionally, only 1 in 1000 potential drugs make it to the clinical trials, with 90% failing the human-testing phase [105]. Drug development is costly and requires substantial investment before pharmaceutical companies can make a profit [106]. The expense in research and development and the long timelines needed for pipelines to produce final products raise increasing concerns about the future prospects of fighting antibiotic resistance.

More than 70% of bacterial pathogens are resistant to at least one current antibiotic treatment [107]. Thus, the need for novel antibiotics or other methods to combat antibiotic resistance is becoming increasingly more dire. Antibiotic-resistant bacterial infections, especially those caused by ESKAPE pathogens (*Enterococcus faecium*, *Staphylococcus aureus*, *Klebsiella pneumoniae*, *Acinetobacter baumannii*, *Pseudomonas aeruginosa*, and *Enterobacter* species), are one of the biggest threats in medicine [108]. Drug-resistant pathogens are projected to surpass cancer as the annual leading cause of death by the end of 2050 [109]. New sources of antibiotics are sought to treat multidrug-resistant (MDR) strains. Through the increase and misuse of antibiotics, antibiotic resistance has been selected for in otherwise easily treatable pathogens. MDR Gram-negative bacteria pose major issues for novel antibiotic development due to resistance mechanisms that are not specific to particular classes of antibiotics [108,110]. Terrestrial *Streptomyces* strains have been isolated that produce bioactive compounds against multiple ESKAPE pathogens [111]. Marine actinomycetes isolated from ocean sediments have been shown to produce antibiotics that are active against a multitude of multidrug-resistant (MDR) bacteria ranging from Gram-negative bacteria, such as carbapenem-resistant Enterobacteria (CRE), to Gram-positive bacteria, such as methicillin-resistant *Staphylococcus aureus* (MRSA) and vancomycin-resistant *Enterococcus* (VRE) [112,113]. In a study on a *Nonomuraea* sp. strain, antibiotic compounds were isolated which not only had activity against CRE and Gram-positive bacteria but also produced products with anti-HIV activity [112]. Marine actinomycetes are an appealing source of antibiotic discovery to combat MDR infectious agents. Marine mycobacteria will be an interesting group to study as new sources of antibiotics, since *Mycobacterium* is a genus notorious for possessing animal pathogens (*Mycobacterium marinum*) [114]. Thus, marine mycobacteria producing novel antimicrobials to address the challenge of MDR is an intriguing idea—a potential silver lining to a dark cloud.

The development of antimicrobial resistance in *Vibrio* sp. negatively impacts the ability to control infectious disease in aquatic organisms and humans. Cholera and vibriosis are common public health concerns regarding *Vibrio* human infections [115]. Numerous studies cite the development of antibiotic resistance in *Vibrio* human infections. From 1979 to 2005, all Ogawa isolates of *Vibrio cholerae* O1 from cholera patients in Bangladesh became resistant to tetracycline, ampicillin, kanamycin, streptomycin, cotrimoxazole, furazolidone, and erythromycin [116]. More than 370 reports of antimicrobial resistance of *V. cholerae* were published in the past decade. An analysis of 443 *V. cholerae* isolates obtained from the stool of diarrheal patients showed that more than 99% were MDR [117]. Between 2014 and 2015, 42 *Vibrio parahaemolyticus* strains isolated from patients in Shanghai showed 100% resistance against ampicillin and streptomycin and at least 90% resistance against cefazolin, kanamycin, and amikacin [118]. A total of 57 *V. parahaemolyticus* isolates from patients in Thailand between 2001 and 2016 showed complete resistance to ampicillin, ciprofloxacin, and norfloxacin in 100%, 12%, and 2% of the isolates, respectively, and intermediate resistance to ciprofloxacin and norfloxacin in over 50% of the isolates [119]. Alternative antimicrobials would aid in the treatment of MDR *Vibrio* pathogens, as well as other diseases that have become resistant to traditional antibiotics.

*Streptomyces* isolates are possible agents to combat MDR *Vibrio* pathogens [113]. In a study on *Streptomyces* strains isolated from sediments of the Caspian Sea, strains of *Streptomyces plicatus* and *Streptomyces enissocaesilis* produced antimicrobial agents that inhibited the growth of MDR *Vibrio harveyi* and *Vibrio proteolyticus*. *Streptomyces enissocaesilis* was also found to inhibit *V. parahaemolyticus* growth. Furthermore, two of the isolates showed antibacterial activity against MRSA and VRE. Purified DOPA melanin produced by *Streptomyces* sp. isolated from the coast of Mumbai, India showed strong antibacterial activity against various fish and human *Vibrio* pathogens, including *V. fluvialis*, *V. splendidus*, and *V. parahaemolyticus* [120]. Similarly, *Litopenaeus vannamei* (whiteleg shrimp) post-larvae benefited from nontoxic diets with *Streptomyces* strains when exposed to *V. parahaemolyticus*, resulting in better weight gain, increases in hepatopancreas health, and higher chances of survival when compared to a control group [121]. Further, two *Streptomyces* isolates from India inhibited *V. cholerae* growth [122] and three actinobacterial crude extracts from Indonesia displayed similar results, with the latter exhibiting prospective characteristics of treatments for anti-biofilm formation and quorum quenching.

While *Streptomyces* is the largest and most common source of natural metabolites within Actinobacteria, there is promising research for other actinobacterial species. One study showed that *Micromonospora* sp. inhibited biofilm formation in *V. cholerae* and quorum sensing pathways governed by luxO and hapR [123], which resulted in the downregulation of other virulence factors (cholera toxin, toxin-coregulated pili, and hemagglutinin/protease A). In another study, a *Bachybacterium* strain suppressed *V. alginolyticus* growth [124]. Exploring different species within Actinobacteria, particularly strains of *Streptomyces*, *Micromonospora*, and *Bachybacterium* are reasonable sources of nontoxic antibiotics to treat infections by *Vibrio*. While many of these studies have focused on *Vibrio* infections in aquatic life, marine actinomycete-derived antibiotics and extracts may still be powerful tools in the future study of anti-*Vibrio* treatments in human medicine. When working with microbial crude extracts, cytotoxicity to the patient should be ruled out, if the goal is the identification of clinical or veterinary antibiotics. Methodologies have been developed for the screening, isolation, identification, and characterization of useful bioactive compounds and secondary metabolites from crude extracts derived from various organisms [125,126]. These protocols include mass spectroscopy, liquid and gas chromatography, nuclear magnetic resonance, and other experimental procedures. These crude extract methodologies can be adapted, modified, and optimized for marine actinomycetes.

### 2.3. Antitumor

There is notable anticancer therapeutic potential for actinomycetes, especially those whose products are associated with minimal side effects compared to conventional chemotherapy, for instance the compound salinosporamide A [127]. Adriamycin, isolated from *Streptomyces peucetius* [2], inhibits DNA replication and is an anticancer drug. Other effective products for cancer chemotherapeutics are actinomycin D, bleomycin, anthracyclines (daunorubicin), and mitosanes (mitomycin C). These drugs were obtained from *Streptomyces verticillus*, *Streptomyces peucetius*, *Streptomyces caespitosus*, and other intrageneric isolates [107]. Marine actinomycin compounds with antitumor potential include streptochlorin, actinofuranones, aureoverticillactam, chalocomycin B, cyanosporasides, komodoquinones, nonactin, resitoflavine, sporolides, tetracenomycin D, thiocoraline, t-muurolol, butenolides, echinosporins, and streptokordin. Important secondary metabolites from marine actinomycetes with antitumor potential include streptopyrrolidine, cyclo-(L-Pro-L-Met), streptochlorin, lynamicins, marizomib, and thiocoraline [128]. Two examples of novel anticancer metabolites are the compound extracts ULDF4 and ULDF5 derived from *Streptomyces* strains found at Lagos. ULDF4 and ULDF5 exhibit cytotoxicity against human acute myelocytic leukemia, cervical carcinoma, human gastric carcinoma, breast adenocarcinoma, and human acute promyelocytic leukemia [129]. ULDF4 and ULDF5 are structurally similar to staurosporine and kigamicin, compounds known for inducing apoptosis and necrosis, respectively. Ketomycin is another prospective antitumor compound. Ketomycin suppressed cellular migration and invasion in breast carcinoma cells, inhibited NF-κB activity used in upstream signaling by hindering the autophosphorylation of IKK-α/IKK-β, and minimized the 3D-invasion of breast carcinoma cells at nontoxic concentrations [130]. Therefore, ketomycin is not only an effective antibiotic, but also a structurally simple antitumor agent for mammalian cells. The search for further antitumor agents also includes the analysis of BGCs and chemotherapeutic gene clusters (CGCs). Complementary anticancer treatments have been discovered in *Streptomyces* via the diverse and variable patterns of the phylogenetic distribution of BGCs and CGCs [131]. These hybrid BGCs and CGCs are prospective sources of novel secondary metabolites and chemotherapeutic agents for pharmaceuticals. The utilization of *Streptomyces* compounds such as staurosporine, kigamicin, and ketomycin as well as BGCs/CGCs should be further investigated for the development of new antitumor treatments.

### 2.4. Anthelmintic Activity

Avermectin, derived from *Streptomyces avermitilis* [132], is a strong anthelmintic agent historically used to target nematodes, arachnids, and insects [133]. Ivermectin, one of the avermectins, is considered a wonder drug for its versatile application in veterinary and human medicine [134]. Ivermectin is used to treat heartworm infection in canines [135], onchocerciasis in humans [136], and malaria transmission in mosquitoes [137]. Abamectin and emamectin benzoate (both avermectins) are common nematocides for pine wilt disease [138]. However, heavy reliance on avermectins may predispose the target to become resistant; therefore, the development of alternative treatments is becoming increasingly popular. In a study on actinomycete isolate V5 (*Streptomyces* sp.) from a rhizosphere soil sample in Tamil Nadu, India, the actinomycete culture filtrate inhibited egg production of *Meloidogyne incognita*, a root knot nematode that infests eggplant [139]. In a similar study on an isolate from Korean forest soil samples, a *Streptomyces* sp. displayed the strongest nematocidal activity of the five thousand types of Actinobacteria screened. A highly effective nematocide from *Streptomyces* sp. was spectinabilin. Spectinabilin inhibited the development of *Bursaphelenchus xylophilus*, an endoparasitic nematode found in pine forests [124]. The rare nitrophenyl-substituted polyketide metabolite is already known for its antimalarial and antiviral characteristics [140]. Spectinabilin is a practical alternative nematocidal agent to abamectin [124]. Actinobacteria indicate beneficial anthelmintic activity and continue to be a viable source of alternative treatments for many applications [124,139].

### 2.5. Biofouling

Biofouling, caused by unwanted biofilm formation, is an industrial corrosion and natural efficiency problem found on the surfaces of medical devices and implants [141], membrane systems [142], ship hulls, pipelines [143], and various other environments. Biofilms are found in human dental plaque, intestinal tracts, implants, tubes, and stents [144]. Such biofilms are responsible for urinary tract infections, cystic fibrosis, chronic obstructive pulmonary disease, and chronic wounds [145]. Marine biofouling settlement damages ship hulls and increases dragging up to 60%, resulting in up to 40% more fuel consumption and increased CO_2_ and SO_2_ pollution [146]. Hull biofouling is also often responsible for the introduction and establishment of non-indigenous marine species [147], presenting further environmental concerns. New methods are needed to improve biofouling prevention and removal, and one of the areas receiving recent attention is the use of anti-biofilm agents derived from marine actinomycetes [148]. Many marine actinomycetes demonstrate anti-biofouling properties. In a study of 40 marine actinomycetes isolated from a soil sample of *Rhizophora apiculata*, *Streptomyces thermolineatus*, *Streptomyces variabilis*, and *Streptomyces althioticus* strains showed the maximum zone of inhibition and higher anti-biofilm activity. The chloroform extract of the *Streptomyces althioticus* strain demonstrated the highest biofilm inhibitory activity against *Psychrobacter celer*, *Psychrobacter alimentarius*, and *Kocuria rhizophila* [149]. Napyradiomycin derivatives of actinomycetes found in the sediments of the Madeira Archipelago demonstrated ≥80% biofilm inhibition of *Marinobacter hydrocarbonoclasticus*, *Micrococcus luteus*, and *Pseudooceanicola batsensis*. Biofilm inhibition was also evident in the settlement of *Mytilus galloprovincialis* larvae without toxicity to the invertebrate. Napyradiomycins are a viable nontoxic source for marine antifouling paints and pipeline coatings [150]. Another study found *Streptomyces* sp. and *Arthrobacter mysorens* extracts had broad spectrum activity against biofouling bacteria [33]. The extracts are practicable alternatives to banned toxic copper oxide and tributyltin oxide anti-fouling coatings [151]. These studies indicate that marine actinomycete metabolites are viable anti-biofilm and anti-biofouling agents, which would be both economically advantageous and environmentally safer than traditional methods. The use of marine actinomycetes for anti-biofouling treatments is a promising area of medical and industrial research.

## 3. Environmental Applications

In addition to their utility in medical contexts, actinomycetes are being evaluated for a variety of environmental applications, including anti-biofouling and the bioremediation of inorganic and organic wastes, metals, and radioactive wastes. Aquatic actinomycetes may be especially useful for these purposes as they preclude the need to adapt terrestrial bacteria to survive in marine or freshwater conditions. Here, we provide a brief synopsis of ongoing bioremediation efforts, focusing primarily on marine actinomycetes and their potential to degrade and prevent pollution.

### 3.1. Pesticide Production

Pesticides are chemical agents used to deter or kill pests, including insects and fungi, and are one of the most widely distributed pollutants in the environment [152]. The agricultural application of synthetic pesticides causes economic, environmental, and biodiversity damages due to the negative effects of water runoff contamination, human illness, high cost of production, and ecosystem deterioration [153]. Fungicides are commonly used to treat fungal diseases, with 80% of plant diseases caused by pathogenic fungi [154]. Established antifungal metabolites derived from actinomycetes include kasugamycin [155], mildiomycin [156], polyoxin B, polyoxin D [157], and validamycin A [2]. These compounds are particularly useful because they are effective at combatting fungi while not harming animals. Kasugamycin does not inhibit protein biosynthesis in mammals and is an effective treatment against rice blast, also known as *Pyricularia oryzae cavara* [155]. Similarly, mildiomycin does not inhibit translation in mammals, only fungal protein biosynthesis [156]. Validamycin A is also an attractive nontoxic antifungal treatment for suppressing the metabolization of trehalose because vertebrates do not depend on this sugar as a primary source of energy [158]. Another effective form of pathogenic regulation is the inhibition of fungal cell wall synthesis by using polyoxin B and D to inhibit chitin synthase [157]. Further discovery of safe and inexpensive pesticides would help offset reliance on synthetic pesticides.

The discovery of new actinomycete isolates is also highly appealing due to their demonstrated ability to treat multiple pathogenic fungal strains. In a study within soil at various locations in Mexico, actinomycete isolates showed high inhibitory potential against phytopathogenic fungi *Rhizoctonia solani*, *Phytophthora capsici*, and *Fusarium oxysporum*. Furthermore, chili plants (*Capsicum annuum*) inoculated with the actinomycete isolates demonstrated increased plant height and fruit weight with respect to the control, possibly due to their production of growth promoters such as indole acetic acid, gibberellins, and cytokinin-like substances [153]. In another study, many of the 23 Actinobacteria isolates from *Odontotermes formosanus* showed inhibition of both the termite cultivar *Termitomyces* and the competitor *Xylaria*. Actinomycin D isolated from *Streptomyces parvulus* exhibited antifungal activity against *Xylaria*, *Magnaporthe grisea*, *Fusarium oxysporum*, *Valsa mali*, *Rhizoctonia solani*, and *Dothiorella gregaria* [159]. Actinomycete isolates show significant promise as a source for antifungal remedies.

### 3.2. Bioremediation

Actinobacteria’s utility in bioremediation is another area of active research, especially as the industrial contamination of soil and water systems is becoming an increasing cause of concern among environmental activists [152]. Better systems are needed for the cleanup of pesticides, metals, and mixed pollution. Currently, methods for the removal of pesticides and other toxic chemical substances from soil and water exist but are not always effective, particularly with inorganic compounds. While organic compounds can be degraded completely into safe products, inorganic compounds are frequently degraded only partially, creating intermediates that may be more toxic than the original pesticide [152]. Other methods, such as the use of bacteria in bioremediation, are being evaluated to circumvent these problems. Actinobacteria are a practicable choice for this purpose, as they are ubiquitous in the soil and water and already perform the function of maintaining ecological balance by degrading both organic and inorganic compounds in their environments [152]. Several species of Actinobacteria have been found to use pesticides as carbon sources, degrading them completely and returning them to nontoxic base elements and compounds [86]. For example, several strains of *Streptomyces* (including *Streptomyces espinosus*) have been found to produce tyrosinase enzymes, which are instrumental in the removal of phenols, a component of many pesticides that pollute water sources. Tyrosinase isolated from these bacteria was more effective than commonly used tyrosinase, which was previously isolated from various mushrooms. Although this is just one example, it demonstrates that Actinobacteria can perform bioremediation in culture and also produce chemicals that can be used separately for the removal of specific pollutants [160]. Marine actinomycetes, including members of the genus *Mycobacterium* [161] and *Streptomyces* [162], have been found to possess the ability to degrade pesticides and other organic pollutants effectively [161,162]. Thus, they may be good candidates for removing organic pollutants from marine environments. Some *Streptomyces* members, including marine *Streptomyces* such as *S. albus chlorinus* [163], have even been found to have properties that allow for effective pesticide/herbicide activity without causing environmental damage [161,163,164,165]. These species should be investigated as new sources of pesticides that would replace current treatments, preventing the need for pesticide removal efforts.

In addition to the remediation and replacement of pesticides, actinomycetes have also been implicated as possible candidates for the removal of toxic metals, which are notoriously difficult to remove from the environment using traditional methods. Many Actinobacteria species are tolerant to heavy metals, in particular members of the genus *Streptomyces* [152]. Since Actinobacteria is one of the only phyla with high metal tolerance [166], the search for bacteria to use in metal bioremediation necessarily must include this phylum and will likely focus on marine members of the order Actinomycetales for applications in the ocean. *Streptomyces* species are of particular interest, since many of them possess both the ability to tolerate large concentrations of toxic metals as well as the unique ability to precipitate, bioaccumulate, and adsorb these metals [167]. In one study, *Streptomyces* species such as *S. lividans* were shown to absorb metals such as Cu (II) and Cd (II) [168]. In another study, both *Streptomyces* and *Amycolatopsis* species were able to bioaccumulate Pb, Cd, Cr, and Zn [169]. The order Actinomycetales has a strong capacity to metabolically process heavy metals, a vital and yet underexplored area that warrants further research and application.

The ability of *Streptomyces* to degrade organic and inorganic pesticides, as well as to process metals in a way that draws them out of the environment, also makes this genus a strong candidate for the bioremediation of mixed pollution [152]. Mixed pollution, wherein different toxic organic and inorganic compounds are present together, is a growing issue. It comprises most environmental pollution and requires multiple remediation solutions to remove all toxins, some of which may inadvertently interact with other pollutants to create new toxic compounds. The specific marine actinomycete species chosen for a bioremediation effort may need to be adjusted on a case-by-case basis. The most prominent strategy to ameliorate this problem is to include mixed cultures, involving several species, to each perform one or many aspects of the cleanup for a given area [152]. This has been investigated as a promising treatment in mixed heavy-metal cleanup as well as in the removal of pollutants that involve both heavy metals and organic/inorganic pesticides [170,171]. Certain actinomycetes such as species of *Rhodococcus* [81] and *Streptomyces* [172] are able to degrade plastics, which are an increasing concern among environmentalists [81,172,173]. Actinomycetes offer much potential here, since many members are able to survive extreme environments and will tolerate highly toxic conditions [152].

Finally, another underexplored area of bioremediation research is the utilization of actinomycetes in the cleanup of radioactive wastes. In a study of marine actinobacteria around a nuclear power plant in India, a species of *Nocardiopsis* was discovered that was able to adsorb significant amounts of radioactive cesium. Since this adsorption was performed through passive mechanisms such as extracellular binding, the adsorption was performed whether the species was alive or dead [174,175]. In another study, marine *Streptomyces* was able to produce an extracellular polymeric substance (EPS) that aided in the biosorption of Sr^2+^ [174]. Likewise, *Streptomyces sporoverrucosus* was found to adsorb uranium by accumulating it on cell walls in high amounts [176]. While this research is still very new, the discovery of actinomycetes capable of removing radioactive waste seems a promising avenue, especially since it would present a much safer, cheaper, and more eco-friendly method than current cleanup practices for radioactive waste [175]. As more research is conducted on the ability of different actinomycetes to adsorb various radioactive species, a similar mixed-culture remediation technique may be developed to better equip nuclear power plants for radioactive waste leakage or accidents.

## 4. Industrial Applications

Members of the Actinomycetales order are also highly regarded in various industrial applications. They are being evaluated for probiotic use in aquaculture, biofuel production, and the production of compounds used in the development of plastics, detergents, and other products. While these applications are still in the research and development phase, it is likely that the use of Actinomycetales in these industrial contexts will become more prevalent in the coming years. Here, we highlight a few of the most promising applications.

### 4.1. Probiotics

With the emergence of MDR bacteria, there has been significant pressure to find stronger alternatives to commonly used antibiotics in the aquaculture industry. Antibiotics have traditionally been used as a water supplement to ensure that animals used in seafood are not infected during the farming process. As previously mentioned, a significant concern in aquaculture is infection with *Vibrio* species, which cause the marine illness vibriosis, particularly MDR species of *V. harveyi, V. parahaemolyticus*, and *V. proteolyticus* [113]. One potential way to circumvent this issue is to use probiotics—microorganisms that are introduced to another organism either to compete with pathogens for resources, improve the organism’s internal or external environment, or facilitate/provide nutritional benefit [177]. Currently, species used as probiotics include *Lactobacillus, Bacillus, Bifidobacterium*, and *Lactococcus* species, and yeasts such as *Saccharomyces cerevisiae*; however, Actinomycetales species, particularly members of the *Streptomyces* genus, have been receiving recent attention as potential new sources of probiotics [178]. *Streptomyces* spp. are strong candidates for probiotic use, mainly because of their ability to produce antibiotics that are active against regular and MDR *Vibrio* species [113,179]. This ability can be enhanced in certain conditions by combining *Streptomyces* with other bacteria. In one study, when shrimp that were infected with a pathogenic *Vibrio* species were treated with a *Streptomyces–Bacillus* combination, hemocyte production increased and survival were improved significantly over groups in which a single species was used [179]. *Streptomyces* can also be used to prevent the growth of harmful bacteria by simply outcompeting them for resources, particularly iron (exploitative competition), which is needed for growth and biofilm formation [180].

*Streptomyces* strains have also been found to produce amylase, lipase, protease, and other enzymes that facilitate digestion and the absorption of nutrients in the host organism, providing an additional benefit that further displays the utility of actinomycetes as probiotics. Moreover, planktonic *Streptomyces* can actually be added to aquaculture feed, particularly live feed such as shrimp, or be used as feed itself for animals that primarily consume planktonic bacteria [178]. Hemolytic and genetic assays of these species show that they appear to be relatively safe for marine animals [113,177,179,181], though concerns do exist regarding the transfer of intrinsic antibiotic resistance genes of *Streptomyces* to other bacteria through horizontal gene transfer. Perhaps using subinhibitory concentrations of antibiotics can be a compromise [101,102]. Some marine actinobacteria can be difficult to culture, which might present a major barrier to their use as probiotics [177]. Some studies have been able to culture specific strains using techniques such as submerged fermentation. An interesting question is how many actinomycetes in terrestrial, freshwater, and marine habitats are entirely nonculturable because researchers are ignorant of their growth requirements [182]. Some research has also reverted to extracting the secondary metabolites that provide antibiotic activity for use as an easier alternative treatment without culturing the bacteria for distribution [113]; however, this is not a permanent solution as it creates a new antibiotic treatment that may generate the same drug resistance issues as current antibiotics. Finally, many marine *Streptomyces* produce the compounds geosmin and 2-methylisoborneol, both of which produce a taste described as “earthy” or “musky” that makes farmed seafood smell and taste less appealing to consumers. Ozonating the water containing *Streptomyces* seems to break down this smell and taste; however, the need to ozonate the water could present another barrier against using *Streptomyces* for many aquaculture farms [178]. Still, the use of *Streptomyces* and other actinomycetes seems to be a promising avenue by which aquaculture methods can be improved.

### 4.2. Biofuels

Rather than simply using them as methods to clean up pollution, some researchers suggest using actinomycetes in ways that would prevent the formation of these pollutants in the first place [183]. In particular, the use of actinomycetes as a possible producer of biofuels has been examined as a promising new area of research. Since actinomycetes, particularly soil isolates, can perform carbon cycling that degrades hydrocarbons and other organic compounds, it appears that actinomycetes would be able to break plant organic matter into sugars that could be used to produce energy [183]. In fact, many actinomycetes, including *Streptomyces, Rhodococcus, Nocardia, Corynebacterium*, and *Mycobacterium*, have been found to have enzymes that degrade carbohydrates into these simple sugars. This degradation allows for the production of compounds such as bioethanol and biodiesel, which can replace current ethanol and diesel-based fuels derived from nonrenewable resources [184]. *Streptomyces,* in particular, is a strong candidate for biofuel production, since there is a lot of research supporting its ability to degrade cellulose and other plant biomass efficiently [185]. This allows for mass-manufacturing of these monosaccharide-based fuels [183]. Thus far, research on such biofuels has been centered on terrestrial actinomycetes, yet there is evidence of marine actinomycetes producing carbohydrate-degrading enzymes such as cellulase and xylanase [186,187,188]. As research continues to explore the use of actinomycetes in biofuel production, marine isolates will be considered as a possible source. This application is still new, but actinomycete species could become significant in the production of biofuels in the near future.

### 4.3. Chemical Additives

The search for actinomycete secondary metabolites has discovered compounds that can be used in other industrial contexts, such as the development of plastics. In one study, *S. lividans* was engineered with phenolic acid decarboxylase derived from other species, allowing for the production of 4-vinylphenol, a plastics additive, from cellulose [172]. In another study, *Streptomyces maritimus* benzoate production was enhanced, allowing it to utilize a novel pathway to directly synthesize benzoic acid from cellulose [189]. As benzoic acid is used in a variety of ways in cosmetics, food preservatives, hygiene products, and pharmaceutical products [190], this discovery could pave the way for more efficiency in these industrial fields. Some of the common enzymes currently utilized in medical applications also serve as detergents, and have been successfully utilized in food and textile production [191]. While this is not an exhaustive list of the potential uses of actinomycetes, it demonstrates the versatility of these microorganisms within industrial spheres. As more marine actinomycetes are discovered and cultured, we expect that their unique properties will be further exploited in these industrial fields.

## 5. Areas of Further Exploration

To aid in the isolation and development of actinomycete products, several actinomycete characteristics should be explored in further detail. Some of these characteristics, discussed here, include their ability to perform quorum sensing, transfer and receive genes through plasmids, form symbiotic relationships, and interact with phages. In combination with genetic engineering and other development techniques, the investigation of these characteristics should provide stronger foundational knowledge of actinomycete function, as well as more efficient product development methodologies.

### 5.1. Incapacitation of Quorum Sensing and Anti-Biofilm Agents Not Affecting Growth

Bacteria use quorum sensing, a form of cell-to-cell chemical communication, for regulating various microbial physiological activities, such as biofilm formation, virulence factor expression, and bioluminescence. In quorum sensing, extracellular signaling molecules, or autoinducers, are produced and released proportionally to cell density. Since quorum sensing is cross-connected to many other physiological networks and regulons in a cell, the disruption of the quorum sensing machinery can become a viable approach to inhibit the biofilm production of pathogenic bacteria, along with other aspects of cellular processes [192]. A recent study isolated eight actinomycetes with anti-quorum sensing activity against *Chromobacterium violaceum*. One isolate demonstrated antibacterial activity against *Bacillus subtilis*, a food spoilage bacterium. The highest inhibition and destruction of biofilm activity was evident by an actinomycete isolate against *Bacillus cereus* as well as *Shewanella putrefaciens* [193]. Similarly, strains of *Streptomyces* have been found with anti-quorum sensing activity against *C. violaceum*. Actinomycin D extracted from a strain of *Streptomyces parvulus* was shown to inhibit the violacein production of *C. violaceum* and inhibited the prodigiosin production of *Serratia proteamaculans*, without affecting bacterial growth in either microbe. The *S. parvulus* strain also inhibited the biofilm production of *Pseudomonas aeruginosa*, *Staphylococcus aureus*, *Micrococcus luteus,* and *Ruegeria* sp. [194].

Other than by the constraint of quorum sensing, actinomycetes could be a source of anti-biofilm agents of pathogens and other troublesome microbes, where cellular growth is not directly targeted. Such anti-biofilm agents would make the evolution of resistance to antimicrobial products less likely, since the selection pressure is “softer”. In one study, metabolites of *Streptomyces californicus* strain ADR1 inhibited biofilm formation in *Staphylococcus aureus* [195]. Among 101 strains of *Streptomyces* and rare actinomycetes collected from sea sediments in the Andaman Sea and the Gulf of Thailand, 10 and 13 strains inhibited at least 60% of the biofilm formation of *Escherichia coli* and *Staphylococcus aureus*, respectively [196]. Most of the strains produced nontoxic anti-biofilm metabolites. Further, some *Streptomyces calidiresistens* strains have been found to produce silver nanoparticles (AgNPs), which display anti-biofilm activity. AgNPs combined with antibiotics exhibited enhanced anti-biofilm activity and required lower doses of AgNPs against bacteria and yeast, therefore reducing the Ag toxicity to mammalian cell lines. Actinomycetes can be utilized to inhibit quorum sensing and biofilm formation while simultaneously evading bacteria-evolved resistance, a problem commonly caused by antibiotic treatments [197].

### 5.2. Plasmids and Gene Sharing

The sharing of genomic content between different microbial species, particularly between different phyla, has been a topic of great interest [198]. One way these genes are shared is through plasmids, mobile genomic fragments that contain genes for non-essential but often beneficial functions. Plasmids can be transferred via prokaryotic conjugation, also known as lateral or horizontal gene transfer [198]. The sharing of actinobacterial genes in particular is an important area of study, since many antibiotic-producing isolates also contain genes involved in resistance to self-produced antibiotics (analogous to toxin–antitoxin modules). These genes are often housed on plasmids and may be transferred to different microorganisms [198]. While this is not usually a concern between members of the same species, it is a larger issue when plasmids are transferred between different species or even different phyla, particularly when the plasmids contain antibiotic resistance genes or transposable elements. Successful transfer between bacterial phyla is much less common than transfer within a single phylum due to the differences in genetic machinery and is especially rare for Actinobacteria, as their linear plasmids are often incompatible with other phyla [198]. However, some bacteria are able to receive the linear actinobacterial plasmids, so this kind of transfer still occurs. An analysis of gene networks between the different bacterial phyla showed that transfers between phyla are commonly responsible for some sharing of antibiotic resistance genes. Actinobacteria tend to share plasmids primarily with Proteobacteria, and this transfer often results in the movement of antibiotic resistance to Gammaproteobacteria species, since the plasmid GC content was particularly high [199].

Additionally, since many actinobacterial plasmids are linear as opposed to the traditional circular shape, the presence of a linear plasmid in other bacteria may indicate a previous bacterial conjugation event with Actinobacteria [198]. These transferred plasmids, when containing antibiotic resistance genes (ARGs), may confer new antimicrobial resistance to other bacteria. Studies have demonstrated this phenomenon in various species of pathogenic Proteobacteria, such as genes conferring resistance to the antibiotics chloramphenicol and lincomycin, which contain sequence signatures known to be part of Actinobacteria. These results suggest that these genes were acquired from actinobacterial species [200]. The movement of plasmids containing ARGs to other microbial species is a growing problem that is contributing to MDR. While not much can be done to prevent these conjugation events, more research is needed into potential interventions to circumvent this dilemma, such as the use of probiotics and other techniques that do not rely on antibiotics. Less is known about the transfer of plasmids to Actinobacteria from other phyla, in part because they are not responsible for any additional antibiotic resistance, leading them to be less well studied. There is some evidence of transposable elements contained in plasmids from other phyla making their way into actinobacterial genomes [199]. The role transposons have in horizontal gene transfer outside of plasmids (i.e., transposition between chromosomes) within marine actinomycetes is still unclear. Several studies have demonstrated that plasmids can be artificially introduced into Actinobacteria via in vitro bacterial mating. Generally, these plasmids are introduced from other actinobacterial species with the intention of measuring stability and transposition of specific loci for genetic engineering [201,202], but the ability to introduce new plasmids into actinobacterial strains opens several avenues for the optimization and application of actinobacterial products in multiple spheres—medical, environmental, or industrial.

### 5.3. Phages

When discussing actinomycetes, it is important to acknowledge the role bacteriophages play both in aiding and impairing actinomycete survival. Phages are an important element of many biospheres, particularly aquatic ones, and are frequently up to 10-fold more abundant in the environment than their prokaryotic hosts [203]. Despite this level of abundance, identifying marine phages has been difficult, particularly in marine bacteria because of the difficulty involved in culturing them in a laboratory setting, and therefore traditional phage identification methods such as direct sequencing do not work. The most effective method of aquatic actinobacterial phage (or actinophage) identification has been using metagenomics to sample from regions of interest and to analyze all the viral genome fragments that are found [203]. In these studies, viral genomes are extracted from the water and aligned with known bacterial and viral DNA and RNA to classify the new phages as well as to assign them to a host. One hallmark of actinophages that has been instrumental in host identification has been the WhiB transcriptional regulator, a gene that is unique to Actinobacteria. While not all actinophages possess a WhiB gene, those that do are able to be automatically identified as actinophages, simplifying the bacteriophage identification process [3,203,204]. Other qualities resembling Actinobacteria, such as high GC DNA or RNA content, may also suggest that a phage infects Actinobacteria [203]; however, these qualities are not typically used in identification as they are less accurate than using alignment scores. Not all Actinobacteria have a high GC content, such as freshwater species. Metagenomics has allowed for the discovery of many new marine actinophages within the last decade and allows for their further study. Still, many marine actinophages, particularly ones with larger genomes, remain undiscovered [3]. Actinophages might be a major source of horizontal gene transfer in marine actinomycetes via transduction [205].

Actinophages have been found to perform several functions within their hosts. Phages may undergo a lytic phase, resulting in cell lysis and death. However, many actinophages can also initiate a latent or lysogenic phase, when protecting their host is advantageous [206]. Some of these temperate phages have been found to confer benefits to their hosts, such as protection from predators via the Trojan horse method. When Actinobacteria containing phages, typically from the G1 group, are consumed by phagocytizing eukaryotes and the host bacteria are digested, phages are released along with several toxins they create [204]. One such toxin is ADP-ribosyltransferase, which is thought to prevent actin polymerization, which causes the eukaryote to die and release the rest of the bacteria it consumed [3]. While this does not have direct benefits for the phage or the original host cell consumed, it does provide the advantage of kin selection, since this allows for the survival of the group containing similar genetic material [204]. Another advantage conferred by phages may be protection against oxidative stress from reactive oxygen species (ROS). Some freshwater phages have been found to produce chemicals that defend against oxidative stress from ROS, either by inhibiting their formation, preventing damage, or promoting repair after damage. These protections are more directly beneficial to the phage infecting each host, since they provide relief against ROS damage to the phage genome contained inside the cell [3]. While many of these host-cell benefits are conjectural rather than confirmed, this area is still being explored and the ways phages benefit their actinomycetal host cells will likely be elucidated in the near future. Phages can also be beneficial through their application in techniques such as genetic modification. With the advent of techniques such as CRISPR/Cas9, the genetic modification of actinobacterial species has been made possible, although the utility of this method has only been successfully demonstrated in a handful of model species thus far. CRISPR/Cas9 activity has been difficult to replicate in other actinobacterial species due to the absence of a replicating plasmid [207], but studies attempting to overcome this issue are ongoing. There will likely be more information about Actinobacteria, CRISPR/Cas9, and other genome editing tools in the coming years.

### 5.4. CRISPR/Cas9

In 2012, Dr. Jennifer Doudna and Emmanuelle Charpentier described CRISPR/Cas9′s gene editing capabilities in vivo. From then, this system has been adapted in various applications to advance research in areas from gene therapy to vaccine development [208,209]. One such underutilized capability is the use of CRISPR/Cas9 gene editing to discover and produce bioactive molecules in actinomycetes [210]. In re-examining previously tested terrestrial actinomycetes using new CRISPR technology, several novel secondary metabolites have been recovered. With this technique, the rare antibiotics thiolactomycin, amicetin, phenanthroviridin, and 5-chloro-3-formylinodle were isolated. The downside of this technique is that it is difficult to develop and find plasmid vectors that will be versatile for all actinomycete species. Plasmid pCMU-4, an adaptable plasmid, was developed to more tightly regulate Cas9 expression. This technique can be used alongside normal antimicrobial compound isolation to increase product output in rare marine actinomycetes [211]. However, more research into actinomycetal plasmids is needed to facilitate better gene editing and plasmid design.

Recent studies have investigated the development of more efficient CRISPR/Cas9 tools for actinomycete research. The screening process for CRISPR use is cumbersome, partly due to the plasmid curing steps involved, which can be time consuming and laborious. To circumvent this problem, a dual-function chromogenic screening-based CRISPR/Cas9 system was developed to increase efficiency and speed [212]. With this development, CRISPR can be utilized more effectively with all actinomycetes, including rare and marine actinomycetes. Since previous methods of genomic editing have been largely inefficient in Actinobacteria [213], the advent and development of CRISPR/Cas9 gene editing has provided an opportunity to continue actinomycete gene exploration. CRISPR/Cas9 editing will be an invaluable technique for investigating marine actinomycetes for useful metabolites.

### 5.5. Symbioses with Invertebrates

Marine invertebrates, such as sponges and corals, have been used as a source of bioactive compounds but, due to their small biomass, they are not reliable sources. Evidence has shown that the microorganisms within these invertebrates might be the true sources of these antimicrobial compounds [214]. Marine invertebrates harbor microbes such as actinomycetes that are known to produce antimicrobial compounds [215], which are housed in the invertebrates’ extra- and intracellular spaces [216]. Sponges, for example, provide a favorable environment for marine microbes. Sea sponges are sessile organisms, which allows for the formation of biofilms. Sponges also contain pores that allow water movement through their body plans, and this provides ample nutrients to their microbial symbionts. Additionally, protection is offered against the oceanic environment [217]. All these factors lead to a healthy microbial community within the sponge holobiome. These interactions are mutualistic, as the microbes benefit the sponge by providing nutrients through secretions and protection against other pathogenic microbes, and by reinforcing the sponge structure [217,218].

Since the 1960s, over 4500 bioactive compounds have been derived from microbes in sponges [219]. In one study, a bioactive compound from a marine actinomycete species (*Nocardiopsis dassonvillei* MAD08) had many antimicrobial properties, including as a surfactant [220]. Surfactants help prevent the adhesion of a pathogen to the host, which is protective against Gram-negative bacteria. Since Gram-negative bacteria have a second cell membrane, many antibiotics have a hard time passing through antibiotic resistance mechanisms such as efflux pumps in the membrane. By targeting a cell surface structure on the outside of the cell, these resistance mechanisms become futile and the Gram-negative pathogen becomes permeable to these antibiotics. Preventing adhesion also hinders biofilms, which are virulence factors in conditions such as pneumonia caused by *P. aeruginosa* [221,222]. In a study that used bycatch discards to isolate bioactive compounds from invertebrates and sediment, the researchers were able to use small subunit rRNA sequencing to obtain isolates (including Actinomycetales members such as *Streptomyces*) that exhibited activity against MRSA as well as *Staphylococcus warneri* [223]. Sponges and other invertebrates may be viable sources for the continued isolation of novel marine actinomycete species (including marine mycobacteria) and products [224].

### 5.6. Symbioses with Vertebrates

While much is known about symbiosis between actinomycetes and invertebrates, less is known about mutualisms between actinomycetes and vertebrates. Several species of actinomycetes, including members of the genera *Streptomyces* [225,226], *Nocardiopsis* [34], *Micromonospora* [227], and *Mycobacterium* [226], have been associated with various marine vertebrate microbiomes. The compounds produced by these host-associated marine actinomycetes may have produced bioactive compounds with antimicrobial, antitumor, or other valuable properties [226,227]. For example, *Streptomyces hygroscopicus*, which produces the antitumor compound halichomycin, has been found in the marine fish *Halichoeres bleekeri* [228]. Female pufferfish have been found which contain tetrodotoxin-producing *N. dassonvillei* in their ovaries, suggesting for the first time that tetrodotoxin commonly associated with pufferfish is produced by Actinobacteria rather than the animal itself [34].

Several *Streptomyces* strains found in the gut microbiomes of marine fish were also found to have antibacterial activity, including against *Vibrio* [226,227]. The populations and composition of these vertebrate-associated actinomycetes may be dependent on environmental parameters, such as salinity [229]. This was suggested in a study examining salmon that experienced a decrease in the relative density of actinomycetes when the fish migrated from freshwater to saltwater conditions. This drop in actinomycete populations within the fish microbiomes was associated exclusively with changes in salinity [229]. This suggests possible changes in actinomycete-derived secondary metabolites due to salinity fluxes, which could have an impact on the antimicrobial substances produced by the bacteria. Antibacterial compound-producing actinomycetes have even been found in mammals, as in a study that found porpoises containing *Micromonospora* strains that had antagonistic activity against *Clostridium difficile* and other pathogenic bacteria [227]. It is likely that these actinomycete–vertebrate symbiotic relationships are common but are understudied because of the relative difficulty associated with isolating host-associated species as opposed to free-living forms. More in-depth studies of these marine vertebrate microbiomes may help yield innovative treatments for marine vibriosis or human diseases.

### 5.7. Symbioses with Marine Plants

As with vertebrate and invertebrate hosts, actinomycetes can associate with marine plants. The microbes present both in and around plants create a biome which can be both beneficial and antagonistic. These different interactions are important as climate change has led to abiotic stress, which can lower a plant’s defenses against pathogenic microbes. Some microbes can cause lesions through exotoxin secretion, though others protect the plant from those pathogens through antibiotic production [2]. These beneficial microbes, which are present in both terrestrial and marine environments, provide another source for antibiotic discovery [230].

Endophytic bacteria compose a rare subset of actinomycetes that are present in the tissues of plants [231]. Little is known about what mutualisms actinomycetes in the ocean might form with marine plants, including seagrasses and mangroves (or even green, red, and brown algae). These associations might be the source of novel interesting secondary metabolites that await discovery. These actinomycetes would utilize compositions of carbohydrates, peptides, and other nutrients that are unique to plants and algae [231]. Terrestrial endophytic actinomycetes (EA) have been shown to produce metabolic compounds that act against MDR bacteria [232]. Marine EA metabolites extracted from an ethyl acetate solvent system showed antibiotic activity against MDR bacteria, such as those that cause urinary tract infections [231]. As marine actinomycete isolation efforts continue, the discovery of novel isolates associated with seagrasses, mangroves, and algae may uncover secondary metabolites that have similar anti-MDR activity. Seagrass meadows may contain actinomycetes that possess useful applications in myriad realms. *Streptomyces* isolated from seagrass have been shown to produce anthraquinone-rich compounds, which have antimicrobial properties [233].

Actinomycetes and plants also interact through the environment, primarily through the soil. These soil dwelling actinomycetes assist plants with nitrogen fixation and nutrient production. The bioactive compounds produced by these actinomycete species are used by the plants for protection, and the bacteria that produce these protective bioactive compounds are known as plant growth promoting bacteria (PGPB). Many of these PGPB are *Streptomyces* species that possess both antifungal and antibacterial properties [234]. Though much of the current research on plant–PGPB symbioses in actinomycetes has focused on terrestrial environments, marine actinomycetes may also display similar growth-promoting properties. Actinomycetes isolated from the soil of the coastal areas of Mauritius, the Bahamas, the Canary Islands, and Sri Lanka have shown antimicrobial activity against Gram-positive bacteria [235]. In sediments surrounding mangrove plants, actinomycetes have been isolated which expressed antitumor and antimicrobial activity in both the marine sediments and the plants themselves [236,237]. The documentation of nitrogen fixation by marine actinomycetes is scant in the scientific literature. Whether symbioses exist between nitrogen fixing marine actinomycetes and marine plants that are analogous to terrestrial *Frankia* and alder trees (*Alnus*) [238] remains an open question.

### 5.8. Zymology, Fermented Foods, and Enology

Zymology, fermented foods, enology, and the production of other alcoholic beverages are other possible (and quite overlooked) applications for marine actinomycetes. Although bacterial growth during brewing and wine making is typically seen as undesirable, since it can cause beer and wine spoilage, bacterial fermentation processes are used alongside yeast (*Saccharomyces cerevisiae*) to create beverages with “alternative” flavors and aromas [239,240]. For instance, *Lactobacillus* and *Pediococcus* are used to introduce a sour or tart taste in beer. *Enterobacter*, *Lactococcus*, and *Leuconostoc* are important constituents in lambic and sour beers, along with wild ales [241]. Moreover, bacteria play an integral part in brewing, where there is secondary fermentation as part of a long aging process. Additionally, lactic acid bacteria can be used in brewing to increase flavor stability or to lower beer viscosity by providing some acidification [241]. A major challenge to breweries and wineries is spoilage due to microbial contamination. A valuable contribution bacteria can have during brewing and wine making is the production of antimicrobial compounds (microbial allelopathy) that would inhibit the growth of unwelcomed microorganisms, which would otherwise ruin precious batches [242]. This definitely is an area where actinomycetes could play a pivotal role, as their secondary metabolites could prevent substantial economic losses to breweries and wineries worldwide. Nisin (a polycyclic antibacterial peptide) is an example of a secondary metabolite that is produced by some bacteria during fermentation that inhibits the growth of contaminating (i.e., allochthonous) microorganisms that would otherwise cause beer and wine spoilage [243]. Nisin is also a preservative of other beverages and foods that are produced with fermentative processes or steps, including kefir, buttermilk, and cottage cheese. Furthermore, nisin-producing bacteria are frequently indigenous (autochthonous) or compatible community members of the processes that are inherent to the production of fermented drinks and foods. Antimicrobial compounds operating against spoilage in fermented drinks and foods can function at subinhibitory concentrations, which can decrease the probability that contaminating microbes will evolve resistance [101,102]. The prevention of contamination and spoilage need not involve allelopathic microbial interactions (interference competition); exploitative competition over a key limiting resource or nutrient (e.g., iron) is another possibility, siderophore secretion, for instance [244]. Actinobacteria have been found to be natural community members in rice wine [245], which can influence flavor [246], and actinomycetes are part of the core microbiota in Chinese Moutai (fermented millet, *Sorghum* spp.) liquor starters [247]. Marine actinomycetes might be especially useful for the production of salty beers (gose) and salt wines (Coan and Turriculae). Actinobacteria have also been isolated from fermented foods [248].

### 5.9. Microbial Metagenomics

With bacteria evolving and accumulating antibiotic resistance faster than scientists can isolate new antibiotics, new techniques need to be developed to help decrease this gap [110]. As previously stated, the search for solutions to the growing MDR problem in medicine necessitates the discovery of new actinomycete products, as well as new methodologies to increase production and yield for these products. Shotgun metagenomics is a technique used to look at the genome composition of multiple organisms. This technique can be used to locate genes known to encode antimicrobial compounds from a group of microorganisms [249]. Moreover, shotgun metagenomics has been used to locate antibiotic resistance genes in actinomycetes derived from sediment samples and microbiomes of macroorganisms. In a study using metagenomics, both actinomycete species and antibiotic biosynthesis pathways were identified, as well as fifty-one different types of antibiotic resistance genes in other microbes that were part of a carp fish’s microbiome [249]. Metagenomics has also been used to isolate secondary metabolite gene clusters. These clusters are highly diverse within actinomycete species as they result from lateral gene transfer. Such exchange events facilitate the movement of important functional clusters between different species, allowing new species to produce these secondary metabolites and providing an evolutionary advantage to the receiving bacteria [49]. Using shotgun metagenomics, these gene clusters can be identified, allowing for future upregulation or in vitro transfer to other microorganisms that would aid in antibiotic development.

A limitation of this technique is that the available metagenomic data in publicly accessible libraries are not complete. Starting a reference library for a heterogeneous sample requires the isolation of environmental DNA from all the species within the sample [250]. This leads to an incomplete genome annotation where up to 50% of the genome is left unknown and assigned hypothetical functions [249]. This is a limitation for marine actinomycetes, as many of them have yet to be discovered and their genomes sequenced. However, significant progress has been made recently [251]. As more marine species are discovered and sequenced, metagenomics should become more accurate and influential for discovering marine actinomycete metabolites.

### 5.10. Microbial Experimental Evolution

There has been little use of microbial experimental evolution with marine actinomycete bacteria. Microbial selection experiments have been conducted with other marine microorganisms with tremendous success to address many basic and applied research questions [252,253]. Microbial experimental evolution could be used with marine actinomycetes for many applications, including within the medical, environmental, and industrial realms. Additionally, microbial experimental evolution with actinomycetes could be combined with bioinformatics (genomics, transcriptomics, and metabolomics) to achieve colossal gains, which has been splendidly accomplished with other microbes [254,255]. This combined strategy of experimental evolution and bioinformatics is especially geared for drug discovery [256,257]. With experimental evolution, microorganisms can be serially passaged under a particular selection regime for hundreds or even thousands of generations [258]. For planktonic growth, these experiments can involve the daily transfer of batch cultures to fresh culture flasks with orbital shaking or the use of continuous cultures in chemostats containing mixing propellers [252]. For biofilm growth, standing (non-shaking) liquid cultures can be utilized [259]. Another method is to periodically transfer microorganisms adhered to glass or plastic beads to new microcosms [260]. Microbial experimental evolution can also be used to study host–microbe interactions [261]. Additionally, the microorganisms evolved in these studies can be placed in the −80 °C freezer at different evolutionary timepoints to create a “frozen fossil record”. The derived populations can later be revived from suspended animation and then be directly competed against the original (“unevolved”) ancestor or other evolutionary timepoints to determine relative fitness within any environment, including the ancestral one or the selection regime [258]. For instance, a marine actinomycete that has undergone evolutionary adaptation within a particular selection regime for 1000 generations could be competed against the 500-generations timepoint or the initial “0 generations” ancestor. These competitions could be performed in the ancestral environment to determine if evolutionary tradeoffs had occurred as a result of adaptation to the novel environment.

## 6. Discussion

The marine members of the order Actinomycetales contain incredible diversity, both in phenotypic/genomic characteristics and utility in various fields. Within the medical field, actinomycetes have been utilized as sources of secondary metabolites that function as antibiotics, antifungals, anthelmintics, and antitumor agents [2]. Autoimmune disorders, allergies, and organ transplant surgeries are other areas where actinomycetes may be the source of beneficial bioactive molecules. For instance, rapamycin (from *Streptomyces hygroscopicus*) helps suppress and prepare the immune system for organ transplants [107]. While current chemotherapeutics mostly originate from terrestrial actinomycetes, marine species are also producers of secondary metabolites and are a promising source of new treatments [48,71]. As more marine actinomycetes are discovered and investigated, particularly rare isolates that are currently understudied, it is expected that more potential antibiotics and other medical treatments will be identified. Furthermore, as more actinomycete plasmids are discovered and analyzed, it is expected that new loci not present in actinomycete chromosomes will be identified as new sources of antibiotics and other drug treatments [67,68]. These advancements will be influential in making progress in the fight against MDR bacteria by serving as a resource for the development of novel antibiotic treatments. We expect that looking to these marine actinomycetes will likely become more commonplace in the search for new antibiotics. Additionally, the use of marine actinomycetes as probiotics has been investigated recently, though research in this field is primarily limited to uses in aquaculture and other aquatic environments [113,177]. While it is unclear whether marine actinomycetes have a place in probiotics given to humans, this is certainly an area where the capacities of other actinomycetes may be explored.

The use of marine actinomycetes may be even more impactful in environmental remediation efforts. Their ability to prevent biofilm formation, thereby preventing biofouling, is key to preventing environmental harm from other traditional anti-biofouling methods [150]. Furthermore, they can be instrumental in the cleanup of harmful chemical wastes such as pesticides [86], toxic metals [168], and radioactive wastes [175]. The ability of many actinomycetes to withstand extreme environments also makes them good candidates for use in mixed pollution, since they can be paired with other actinomycetes or other bacteria to degrade several different types of chemical waste. In these areas, marine actinomycetes have an advantage over terrestrial and other actinomycetes, since many of these sources of pollution either begin or end up in bodies of water [152]. It is likely that the use of marine actinomycetes will continue to expand in these areas, particularly as new planktonic actinomycetes are discovered.

**Figure 1:**
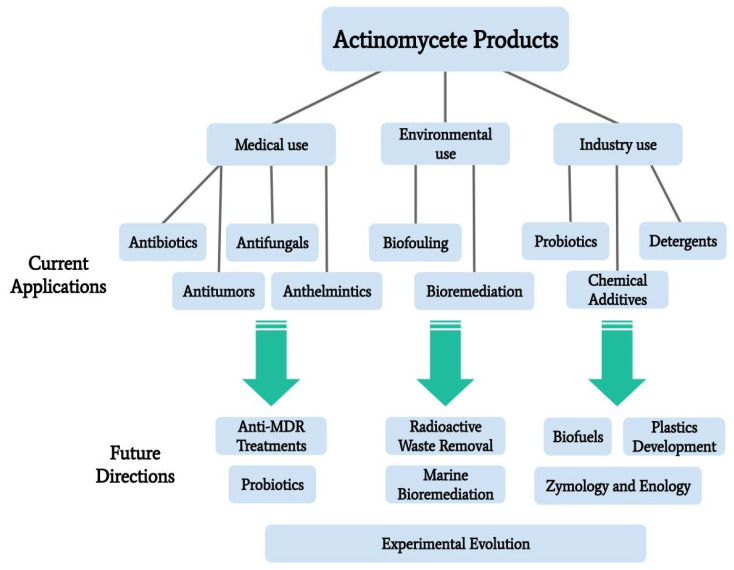
Summary of major applications of marine actinomycetes and suggestions for future directions.

In addition to their use in medical and environmental applications, marine actinomycetes are currently being explored for several different industrial capacities. Though many of these industrial uses are likely confidential as they are still being investigated, a few of these uses include their utility as probiotics in aquaculture, their ability to produce biofuels, and their ability to produce secondary metabolites for use in a variety of other manufacturing contexts [152]. As traditional fossil fuels increase in cost and their impact on the environment gains more attention, the development of biofuels has garnered more traction. The ability of many marine actinomycetes to degrade organic compounds such as hydrocarbons is applicable to the search for new biofuel sources, making several of these species strong contenders for biofuel production [183]. To complement the exploration of probiotics in the medical sphere, marine actinomycetes have also been viewed as promising resources for aquaculture probiotics. This would mitigate the need for antibiotic usage in seafood farming practices and reduce the potential for more bacteria to develop antibiotic resistance [177,178]. Secondary metabolites produced by actinomycetes also demonstrate utility in a variety of cosmetic, agricultural, textile, and plastics-development applications. Although these applications are widespread, the usage of marine actinomycetes for the development of valuable secondary metabolites is still understudied. Marine actinomycetes are becoming more influential in these different fields, and have the potential to revolutionize medical, environmental restoration, and industrial efforts. Figure 1 summarizes many of these applications.

Future study should also look to the exploration of unique actinomycete properties to improve the production of usable secondary metabolites. Through the study of characteristics such as quorum sensing, plasmid biology, symbioses, and interactions with actinophages, secondary metabolite identification and mass production can be streamlined. Harnessing these natural processes, in combination with experimental evolution and genetic engineering to enhance pipeline production, will provide a strong foundation for building more sustainable solutions to issues plaguing environmental, medical, and industrial fields. As more becomes known about their unique properties, it is likely that marine actinomycetes will become just as ubiquitous in applied research as they are in the world’s oceans.
